# Effect of conventional warm-up versus stretching warm-up on physical performance in children soccer players: a randomized crossover trial

**DOI:** 10.3389/fphys.2026.1827385

**Published:** 2026-04-22

**Authors:** Jordan Hernandez-Martínez, Izham Cid-Calfucura, Joaquín Perez-Carcamo, Sebastián Canales-Canales, Eduardo Guzmán-Muñoz, Edgar Vásquez-Carrasco, Tomás Herrera-Valenzuela, Pablo Valdés-Badilla

**Affiliations:** 1 Department of Physical Activity Sciences, Universidad de Los Lagos, Osorno, Chile; 2 Department of Education, Faculty of Humanities, Universidad de la Serena, La Serena, Chile; 3 Department of Physical Activity, Sports and Health Sciences, Faculty of Medical Sciences, Universidad de Santiago de Chile (USACH), Santiago, Chile; 4 Escuela de Kinesiología, Facultad de Salud. Universidad Santo Tomás, Talca, Chile; 5 Escuela de Pedagogía en Educación Física, Facultad de Educación, Universidad Autónoma de Chile, Talca, Chile; 6 School of Occupational Therapy, Faculty of Psychology, Universidad de Talca, Talca, Chile; 7 Centro de Investigación en Ciencias Cognitivas, Faculty of Psychology, Univer-sidad de Talca, Talca, Chile; 8 VITALIS Longevity Center, Universidad de Talca, Talca, Chile; 9 Department of Physical Activity Sciences, Faculty of Education Sciences, Universidad Católica del Maule, Talca, Chile; 10 Sports Coach Career, Faculty of Life Sciences, Universidad Viña del Mar, Viña del Mar, Chile

**Keywords:** athletic performance, exercise, physical fitness, sports, team sports

## Abstract

**Introduction:**

To evaluate the effects of the conventional warm-up (CC) compared to warm-ups that included static (SSC), dynamic (DSC), or ballistic (BSC) stretching on jump performance (CMJ, SJ, DJ), curve sprint speed, agility (ICODT), and ball kicking speed in male children soccer players.

**Methods:**

Eighteen male soccer players (mean age: 11.2 ± 2.4 years) experiencing four warm-up conditions: CC, SSC, DSC, and BSC. They were performed in a random sequence with a 72-hour recovery period in between. After each warm-up, physical performance was measured through the CMJ, SJ, DJ, curved sprint speed, ICODT, and ball kicking speed.

**Results:**

Significant improvements were observed in the ICODT for SSC and DSC compared to CC (p < 0.001). In the CC and SSC conditions, better performance was obtained in curved sprint speed (p = 0.003) compared to DSC, in ball kicking speed with the dominant foot for all stretching conditions (SSC, DSC, and BSC) compared to CC (p < 0.001), and in ball kicking speed with the non-dominant foot for DSC and BSC compared to CC (p = 0.002).

**Discussion:**

In conclusion, that warm-ups incorporating SSC and DSC enhance ICODT in children’s soccer players, while all stretching modalities improve ball kicking speed with the dominant foot compared to a CC. For curve sprint speed, the CC and SSC were more effective than DSC.

## 1 Introduction

Running, jumping, and ball kicking are intermittent, high-intensity actions that are crucial for success in soccer (Dolci et al., 2020). The propose of these actions is either to score or to prevent a goal (Dolci et al., 2020). For instance, a substantial proportion of goals are preceded by straight-line sprinting actions (45%), while vertical jumping actions account for roughly 16% (Emmonds et al., 2019; Faude et al., 2012; González-Rodenas et al., 2020). These actions are strongly associated with high levels of muscle strength, power, and sprint ability, which are determining factors for performance in soccer players across different levels (amateur, professional, elite) and age groups (children, youth, and adults) (Emmonds et al., 2019; González-Rodenas et al., 2020). Considering the decisive role of explosive movements during match play, the implementation of strategies aimed at optimizing physical performance, such as warm-up protocols, becomes crucial (Hammami et al., 2018). The literature consistently reports that different warm-up strategies are effective for enhancing physical readiness for soccer-specific actions (Hammami et al., 2018). Warm-ups typically include high-intensity and short-duration activities aimed at improving performance by increasing intramuscular temperature, nerve conduction velocity, and metabolic reactions (Hammami et al., 2018; Yildiz et al., 2026). It has been well documented that muscle performance can acutely improve by 3.46%–4.21% in strength, and by 1%–20% in sprint and jump ability, following specific warm-ups in adult soccer players (Hammami et al., 2018; Isla et al., 2021).

It has been shown that incorporating static (SSC), dynamic (DSC), or ballistic (BSC) stretching exercises, or proprioceptive neuromuscular facilitation, into conventional warm-ups reduces the incidence of injuries, accelerates recovery, and improves specific physical performance in both static and dynamic balance, as well as lower-body strength (Gil et al., 2019; Hammami et al., 2018; Yıldız and Çebi, 2025). However, the effectiveness of these modalities depends on competitive level, as well as the volume and intensity of stretching applied (López Mariscal et al., 2021). For example, López Mariscal et al. (2021) compared warm-ups including SSC and DSC at different volumes (<10 s and >10 s) and found that both protocols improved performance in linear sprint, countermovement jump (CMJ), and squat jump (SJ) in U16 soccer players and professional adults. Similarly, Vasileiou et al. (2013) reported that DSC significantly improved 20 m maximal sprint performance (p < 0.05), while SSC resulted in decreases of 0.02%–0.06% in amateur players. Gelen (2010) also demonstrated that DSC significantly increased (p < 0.05) ball kicking speed by 3.3% (p < 0.05), whereas SSC reduced it by 2.1% (p < 0.05) in professional players. In contrast, Oliveira et al. (2018) found decreases in CMJ after warm-ups including SSC (−2.3%), DSC (−0.4%), and BSC (−0.7%), as well as declines in 10 m (0.01%–0.04%) and 20 m (0.01%–0.06%) sprint in youth soccer players, compared to a control group performing a conventional warm-up without stretching. More recently, Hernandez-Martinez et al. (2023) examined this topic in Chilean youth soccer players using a randomized experimental design and observed no significant differences (p > 0.05) in sport-specific performance outcomes, including CMJ, linear sprint performance (10–30 m), and ball kicking speed, across SSC-, DSC-, BSC-based warm-ups and a conventional protocol.

Although warm-up optimization is widely recognized as a key factor influencing soccer-specific performance variables such as jumping ability, sprinting capacity, and ball kicking velocity most of the existing evidence has been derived from youth and adult populations, with comparatively scarce data available in children (Gelen, 2010; Hernandez-Martinez et al., 2023; López Mariscal et al., 2021; Oliveira et al., 2018; Vasileiou et al., 2013). Therefore, this study aimed to assess and compare the effects of a conventional warm-up, warm-ups including SSC, DSC, and BSC on jump performance (CMJ, SJ, and drop jump: DJ), curve sprint speed, Illinois Change of Direction Test (ICODT), and ball kicking speed in male children soccer players. We hypothesized that stretching-based warm-up conditions would produce superior performance in several physical performance outcomes compared to a conventional warm-up; however, given the task-specific and developmental characteristics of children, differential responses across performance tasks were expected.

## 2 Methods

### 2.1 Study design

Participant allocation was generated through an online randomization tool (Research Randomizer; (https://www.randomizer.org; accessed on 17 January 2025), with allocation concealment achieved via sealed, opaque envelopes. The study adhered to CONSORT reporting standards (Turner et al., 2012), and the protocol was prospectively registered on ClinicalTrials.gov (NCT06780020; registration date: 12 January 2025).

**Figure 1:**
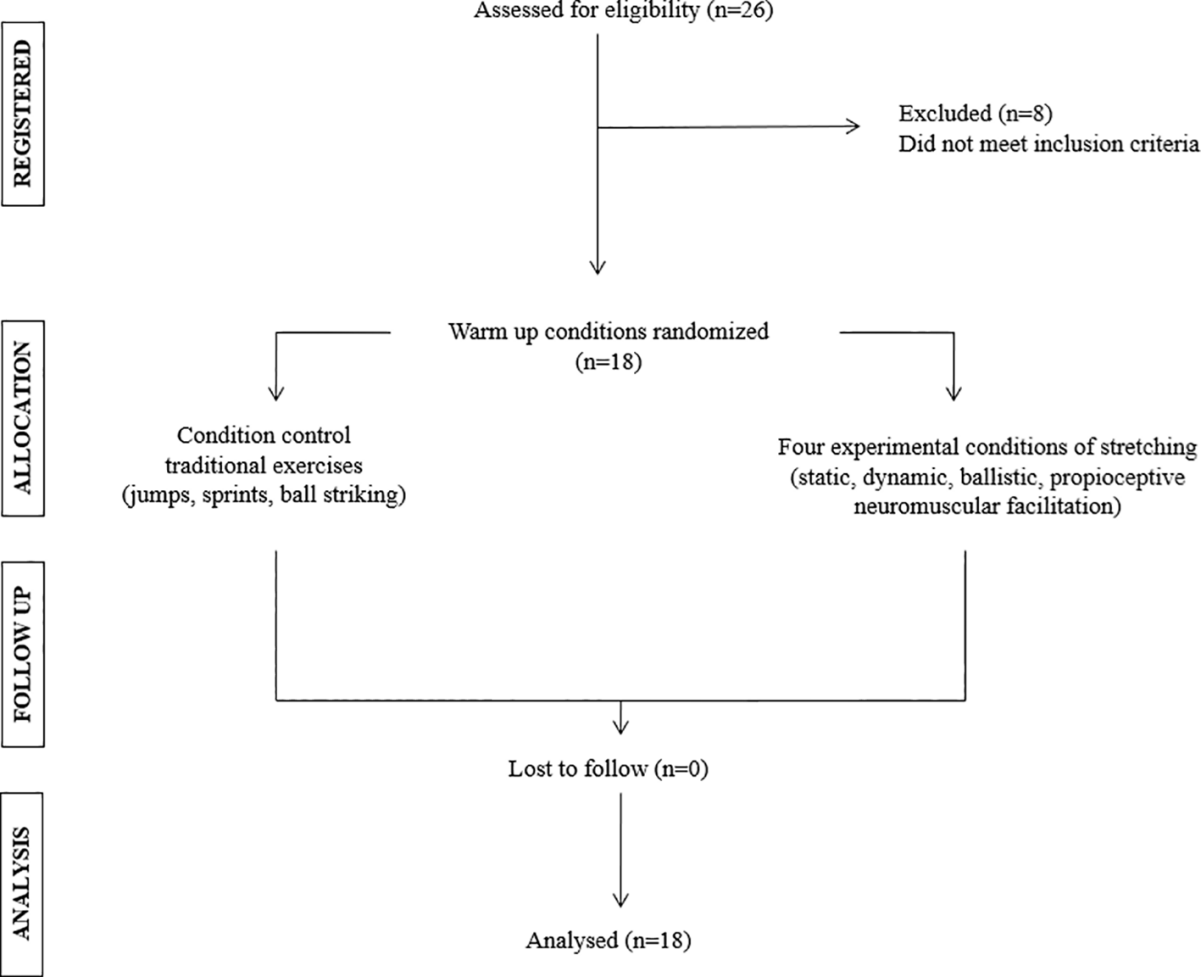
Flowchart of the recruitment process.

A randomized crossover trial and organized homogeneously, in which a group of male soccer players participated in four warm-up conditions, one control condition (CC: conventional warm-up, i.e., no flexibility exercises), and three experimental conditions with stretching exercises (SSC: static, DSC: dynamic, and BSC: ballistic). Participants performed all random warm-up conditions with a 72-hour rest period between them. Anthropometric assessments were carried out in a laboratory under controlled conditions on an empty stomach during the morning with an ambient temperature between 20° and 22° by a qualified professional. Physical performance (jump performance, curved sprint speed, agility, and ball kicking speed) measurements were carried out on a covered synthetic grass playing field to be specific to the terrain where matches are usually played during the afternoon. The clothing used was a T-shirt, shorts, and socks with the soccer shoes usually worn, with an ambient condition of 40% to 50% humidity and an ambient temperature of approximately 20 °C. These assessments were performed by a qualified professional external to this study. The process of selecting participants and randomizing the warm-up conditions is summarized in **Figure 1**.

### 2.2 Participants

An *a priori* power analysis indicated that a minimum of 17 participants per condition was required, informed by effect sizes reported in previous research (Turki et al., 2020). Statistical assumptions included an alpha level of 0.05, 85% power, and a medium standardized effect (d = 0.50). Power calculations were conducted using G*Power software (version 3.1.9.6; Franz Faul, Universität Kiel, Germany). Eighteen male soccer players (age: 11.2 ± 2.4 years; body weight: 47.0 ± 4.3 kg; height: 1.45 ± 3.2 m; body mass index, BMI: 22.4 ± 2.3 kg/m^2^) from an amateur club soccer school in Chile took part. The criteria for inclusion were: (i) absence of injuries that would hinder their ability to complete the warm-up and physical performance evaluations; (ii) wearing suitable athletic attire for the procedures; (iii) not being a member of other soccer schools or teams associated with the current one; (iv) not competing on the same days as the warm-up procedures took place.

Each participant, as well as their legal guardians or proxies, had to sign an informed consent form and an assent form approving the use of the data for scientific reasons. The Bioethics Committee of Universidad Santo Tomás, Chile, examined and approved the research protocol (approval number: EXP-24-78), which was created in accordance with the 2013 Helsinki Declaration (World Medical Association, 2013), regulating research involving human participants. An initial discrepancy in the reported body weight, height, and body mass index (BMI) values was identified as a data transcription error during manuscript preparation. All anthropometric data were subsequently verified against the original measurement records, the descriptive values were corrected accordingly, and all statistical analysis were re-run using the verified dataset, confirming that the results and conclusions of the study remained unchanged.

### 2.3 Anthropometric measurements

The Frankfort plane was used to measure bipedal height in a horizontal posture while a tape measure (Bodymeter 206, SECA, Germany; accuracy to 0.1 cm) was fixed to the wall. An electronic scale (Omron HBF 514, Osaka, Japan; accuracy to 0.1 kg) was used to measure body mass, and bipedal height squared (kg/m^2^) was used to determine BMI. The International Society for the Advancement in Kinanthropometry’s (ISAK) guidelines were followed for all measures (Marfell-Jones et al., 2012).

### 2.4 Jump performance

All jump tests were conducted following prior guidelines (Bosco et al., 1983). For the CMJ, soccer athletes executed maximal effort jumps on a mobile contact platform (ErgoTest, Codogne, Italy) from Ergojump^®^ while keeping their arms positioned on their iliac crests. Takeoff and landing occurred at the precise spot, and players executed complete knee and ankle extensions while in the air. For the SJ, athletes positioned themselves on the contact platform with arms resting on the iliac crest and knees bent at a 90° angle upon receiving the “stop” signal; the athlete held this backward posture while executing the maximum jump. Takeoff and landing were regulated at the precise spot, and athletes executed complete knee and ankle extensions throughout the flight phase. In the DJ test, participants were directed to reduce ground contact time to less than 250 ms after landing from a 20 cm box (Fransz et al., 2018; Hernandez-Martinez et al., 2023). The highest score from three jumps (with a 1-minute break in between each try) was noted for CMJ, SJ, and DJ (Cinemre et al., 2020).

### 2.5 Curve sprint speed

Sprint time was assessed to the nearest 0.01s using single-beam timing gates Brower^®^ Timing System, (Salt Lake City, Utah, United States of America). The test consisted of placing a “semicircle” at a distance of 17 m outside the large area of the soccer field, which is standardized as follows: a radius of 9.15 m from the penalty spot, a distance of 14.6 m from the starting point to the end in a straight line, an angle of 105.84° measured from the penalty spot, and a total distance of 17 m (determined by trigonometric analysis) (Fílter et al., 2020). Photocells were installed at both ends of the semicircle to determine the time it took the players to run the curve. The test started from the left side, and the participant had to run at maximum speed until crossing the finish line. Two attempts were evaluated in both directions of the curve (left and right), recording the results as “dominant side” and “non-dominant side,” depending on the dominance of the children’s soccer players’ lower extremities (Fílter et al., 2020; Pancar et al., 2025).

### 2.6 Agility

The layout and movement pattern of the ICODT were configured according to previously described procedures (Amiri-Khorasani et al., 2010). The test area was delimited using four cones to define a 10 × 5 m rectangle, with an additional set of four markers positioned centrally and spaced 3.3 m apart. Participants began the test from a prone starting position, with the chin in contact with the ground at the start line. Upon initiation, they sprinted forward for 10 m, performed a turn, returned to the start line, navigated through the central markers in a zigzag pattern, and then completed a final 10 m sprint to conclude the test. Athletes were instructed to circumnavigate the markers rather than pass directly over them. Trials were discontinued and repeated after a 3-minute recovery period if instructions were not followed (Amiri-Khorasani et al., 2010). Performance times were recorded using single-beam timing gates (Brower^®^ Timing System, Salt Lake City, UT, USA).

### 2.7 Ball kicking speed

This outcome was assessed after a standardized two-step approach using a size five soccer ball (Molten Vantaggio 5000^®^, FIFA PRO approved). Participants performed maximal instep kicks with both dominant and non-dominant limbs, while ball speed was recorded using a radar device (SR3600, Sports Radar^®^, Homosassa, FL, USA). Three trials were completed per limb with one-minute rest intervals, and the highest value obtained was retained for analysis (Ramírez-Campillo et al., 2015). The kicking speed measurements demonstrated high reliability (ICC = 0.92).

### 2.8 Intervention (warm-up conditions)

The warm-up intervention was designed in accordance with procedures previously applied in adolescent soccer players from Chile (Hernandez-Martinez et al., 2023). Participants assigned to the control condition completed a traditional soccer warm-up lasting 10 minutes, which included approximately 4 minutes of multidirectional jogging performed at moderate-to-vigorous intensities, monitored using the 10-point rating of perceived exertion (RPE) scale (Borg, 1982). Exercise intensity progressed from values between 3 and 5 to levels ranging from 6 to 8. This phase was followed by soccer-specific movements (e.g., jumping actions, ball kicking, and changes of direction), organized into three 60-second bouts interspersed with 60-second recovery periods. No flexibility exercises were included in this condition.

The SSC consisted of a 10-minute warm-up incorporating four exercises targeting the main lower-limb muscle groups (quadriceps, gluteus, hamstrings, and triceps surae). Each exercise was performed in two 30-second repetitions, separated by a 45-second rest interval, with the aim of progressively increasing joint range of motion. Perceived intensity, monitored using the RPE scale, increased from values between 3 and 5 to levels ranging from 6 to 8.

The DSC consisted of a 10-minute dynamic stretching warm-up comprising four exercises targeting the primary lower-limb muscle groups (quadriceps, gluteus, hamstrings, and triceps surae). Each exercise was completed in two 30-second bouts, separated by 45 seconds of rest, and involved oscillatory dynamic movements performed at progressively increasing speeds. Perceived exertion increased from low-to-moderate levels (RPE 3–5) at the beginning to moderate-to-high levels (RPE 6–8) at the end of the protocol.

**Figure 2:**
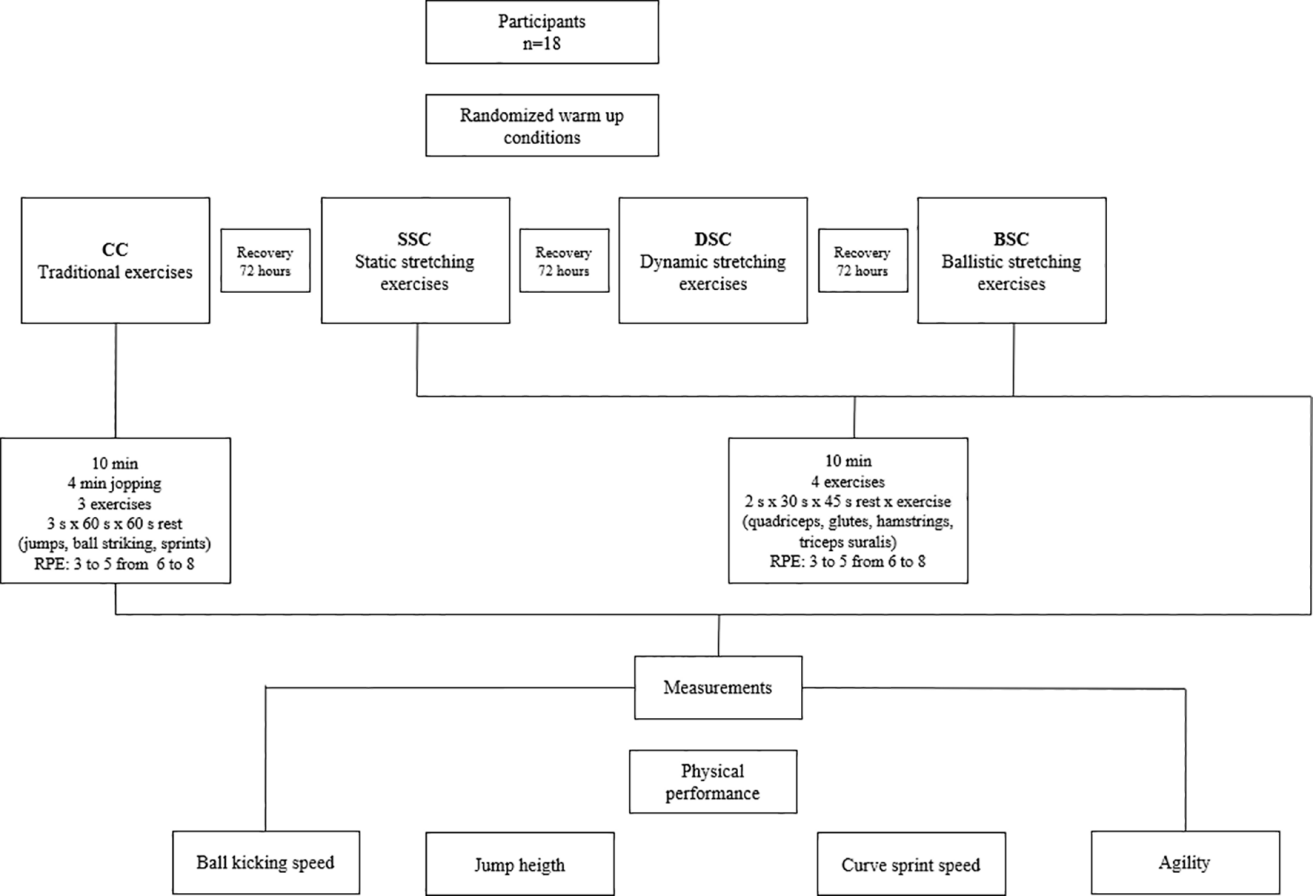
Study design. CC, control condition, conventional exercises (movements in various directions with increasing speeds); SSC, static stretching conditions and exercises (static stretching motions with increasing amplitude); DSC, stands for dynamic stretching condition and dynamic stretching exercises, which involve oscillatory stretching motions with increasing speeds; BSC, ballistic stretching condition, ballistic stretching exercise (maintain an extended position for five seconds, then oscillate for five seconds in progressive increments for thirty seconds); RPE, rating perceived exertion.

The BSC included a ten-minute ballistic stretching warm-up. Four stretching exercises were performed, one for each of the lower body muscle groups (quadriceps, gluteus, hamstrings, and triceps surae). The exercises were divided into two series of 30 seconds each, with a 45-second break in between. Each series involved maintaining an elongated position for five seconds, followed by five seconds of oscillation with progressive increments until the thirty seconds of each series were completed. The intensity peaked between 6 and 8 RPE points, having begun between 3 and 5 points. This warm-up program is shown in **Figure 2**.

### 2.9 Statistical analysis

The mean ± standard deviation was used to report the values. The data’s normality was assessed using the Shapiro-Wilk test, and the homogeneity of variance was assessed using Levene’s test. All of the data showed a normal distribution. ANOVA testing was performed using a mixed model with Bonferroni *post hoc* testing to compare physical performance variables according to the type of warm-up condition. Cohen’s d (Cohen, 1992) was used to calculate the effect size (ES), taking into account a small (0.20–0.49), moderate (0.50–0.79), or large (>0.80) effect. The formula used was d= (M1-M2)/SD (Rendón-Macías et al., 2021). A significance level of p < 0.05 was established in every instance. The statistical analysis was carried out using the SPSS software version 26.

## 3 Results

The analysis revealed no significant differences in jump performance across warm-up conditions for CMJ (F _(3, 32)_ = 1.72; p = 0.18), SJ (F _(3, 32)_ = 0.55; p = 0.64), and DJ (F _(3, 32)_ = 0.68; p = 0.56). In contrast, statistically significant differences were observed in agility, curve sprint speed, and ball kicking speed (p < 0.01). The ICODT showed a significant main effect (F _(3,32)_ = 22.4; p < 0.001) with large effect sizes (ES up to 7.12) favoring DSC and SSC over CC. Regarding curve sprint speed, significant differences were found (F _(3,32)_ = 4.71; p = 0.008) in favor of CC and SSC, compared to DSC, with large effects (ES = 1.27 to 1.70). The ball kicking speed showed major enhancements in the case of the dominant foot (F _(3, 32)_ = 32.6; p < 0.001; ES up to 6.80) as well as the non-dominant foot (F _(3,32)_ = 10.01; p < 0.001; ES up to 2.45) which was mostly after the performance of DSC and BSC. Full descriptive statistics are shown in **Table 1** and detailed pairwise comparisons can be found in **Table 2**.

**Table 1:** Physical performance of children male soccer players according to warm-up conditions.

Soccer players (n = 18)	CC (n = 18)	SSC (n = 18)	DSC (n = 18)	BSC (n = 18)	F value	p value
CMJ (cm)	21.2 ± 5.24	23.8 ± 2.80	25.4 ± 3.35	24.9 ± 4.12	1.72	0.18
SJ (cm)	23.7 ± 3.37	23.1 ± 2.47	24.2 ± 4.1	25.3 ± 4.5	0.55	0.64
DJ (cm)	22.0 ± 3.86	22.5 ± 3.05	23.9 ± 3.42	24.3 ± 4.0	0.68	0.56
ICODT (s)	20.2 ± 0.89	16.5 ± 1.45	15.4 ± 0.34	19.6 ± 2.39	22.4	**< 0.001**
Curve sprint speed (s)	3.44 ± 0.25	3.61 ± 0.20	4.02 ± 0.41	3.64 ± 0.30	4.71	**0.008**
Ball kicking speed of the dominant foot (km/h)	45.5 ± 2.92	65.5 ± 8.76	69.5 ± 4.97	65 ± 4.21	32.6	**0.00**
Ball kicking speed of the non-dominant foot (km/h)	33.8 ± 2.40	42.8 ± 10.3	52.3 ± 4.38	53.4 ± 12.0	10.01	**< 0.001**

CC, control condition; SSC, static stretching condition; DSC, dynamic stretching condition; BSC, ballistic stretching condition; ICODT, Illinois Change of Direction Test. Values in bold indicate statistically significant differences (p < 0.05).

**Table 2:** Differences between intervention conditions of children male soccer players.

Soccer players (n = 18)	CMJ (cm)	SJ (cm)	DJ (cm)	ICODT (s)	Curve sprint speed (s)	Ball kicking speed of the dominant foot (km/h)	Ball kicking speed of the non-dominant foot (km/h)
CC vs. SSC	*d = 0.61^c^* 12.26%	*d = 0.20^b^* 2.53%	*d = 0.14^a^* 2.72%	*d =* 3.07^d^ 18.31%	*d = 0.75^c^* 4.94%	*d = 3.06* ^d^ 43.95%	*d = 1.20* ^d^ 26.67%
CC vs. DSC	*d = 0.95* ^d^ 19.81%	*d = 0.13 ^a^* 2.10%	*d = 0.45 ^b^* 8.63%	*d = 7.12* ^d^ 23.7%	*d = 1.70* ^d^ 16.86%	*d = 5.88* ^d^ *52.74%*	*d = 5.23* ^d^ *54.73%*
CC vs. BSC	*d = 0.78 ^c^* 17.45%	*d = 0.40 ^b^* 6.75%	*d = 0.58 ^c^* *10.45%*	*d = 0.33 ^b^* *2.97%*	*d = 0.72 ^c^* 5.81%	*d = 5.38* ^d^ 42.85%	*d = 2.26* ^d^ 57.98%
SSC vs. DSC	*d = 0.51 ^c^* 6.72%	*d = 0.32 ^b^* 4.72%	*d = 0.43 ^b^* 6.22%	*d = 1.04* ^d^ *6.66%*	*d = 1.27* ^d^ 11.35%	*d = 0.56 ^c^* 6.10%	*d = 1.20* ^d^ *22.19%*
SSC vs. BSC	*d = 0.31 ^b^* *4.62%*	*d = 0.60 ^c^* *9.52%*	*d = 0.50 ^c^* 8.00%	*d = 1.56* ^d^ *18.78%*	*d = 0.11 ^a^* 0.83%	*d = 0.07 ^a^* *0.76%*	*d = 0.94* ^d^ 24.76%
DSC vs. BSC	*d = 0.13 ^a^* 1.96%	*d = 0.25 ^b^* 4.54%	*d = 0.10 ^a^* *1.67%*	*d = 2.46* ^d^ *27.27%*	*d = 1.05* ^d^ *9.45%*	*d = 0.97* ^d^ 6.47%	*d = 0.12 ^a^* *2.10%*

CMJ, countermovement jump; SJ; squat jump; DJ, drop jump; ICODT, Illinois Change of Direction Test; CC, control condition; SSC, static stretching condition; DSC, dynamic stretching condition; BSC, ballistic stretching condition; d, effect size. ^a^Insignificant effect (<0.20). ^b^small effect (0.20–0.49). ^c^Moderate effect (0.50–0.79). ^d^large effect (≥0.80).

**Figure 3:**
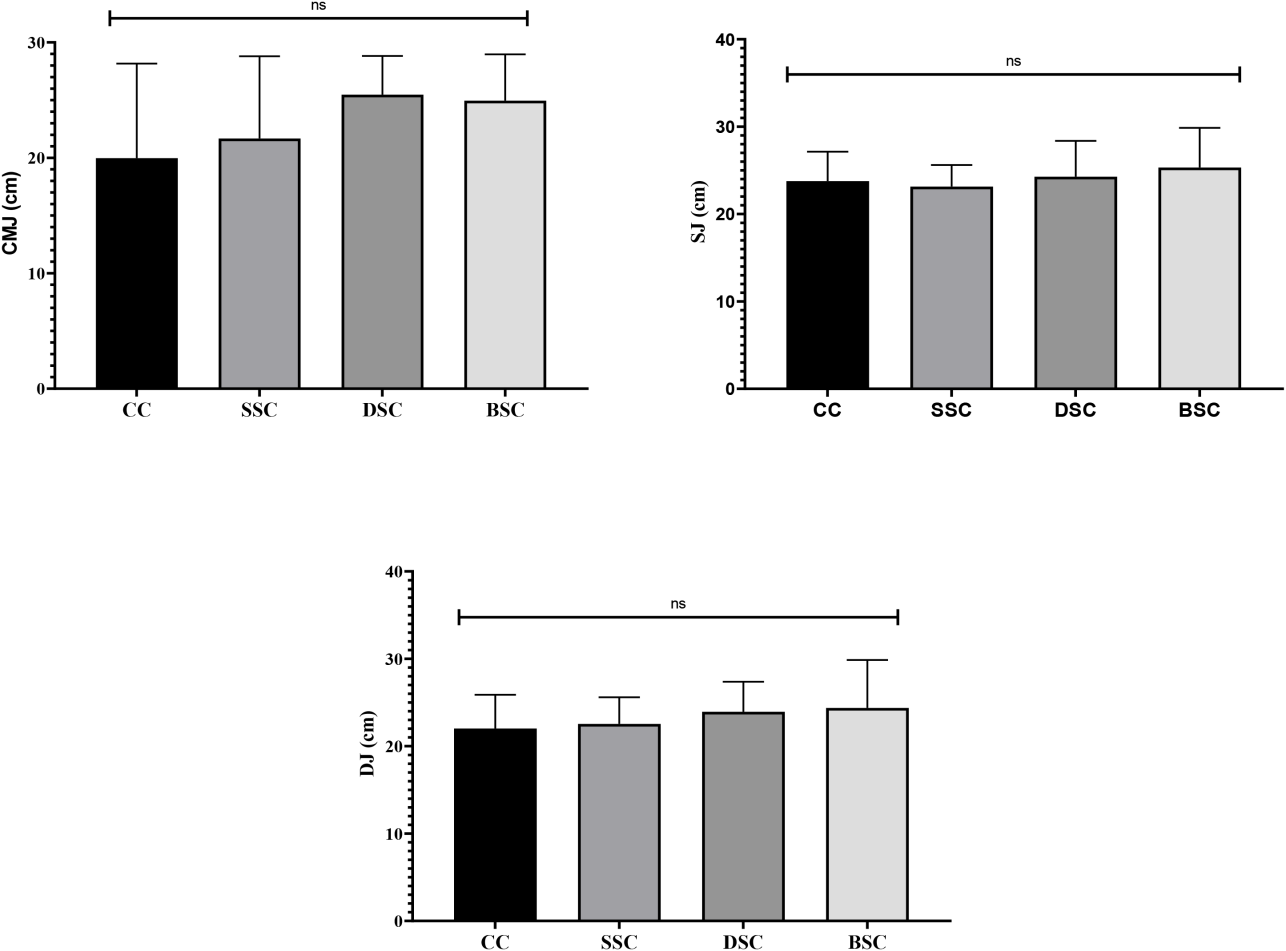
Jump performance depending on warm-up conditions. CMJ, countermovement jump; SJ, squat jump; DJ, drop jump; CC, control condition; SSC, static stretching condition; DSC, dynamic stretching condition; BSC, ballistic stretching condition; ns, no significant.

In the jump performance (CMJ, SJ, and DJ), there were no statistically significant differences in performance between the different warm-up conditions (CMJ: p = 0.13; SJ: p = 0.64; DJ: p = 0.56). However, although the conventional statistical significance threshold (p < 0.05) was not reached, effect sizes ranging from small to large were observed, suggesting a potentially relevant improvement for sports practice, analysis revealed improvement trends in favor of DSC and BSC compared to CC. In CMJ, the increases were moderate to large. These results are presented graphically in **Figure 3**.

**Figure 4:**
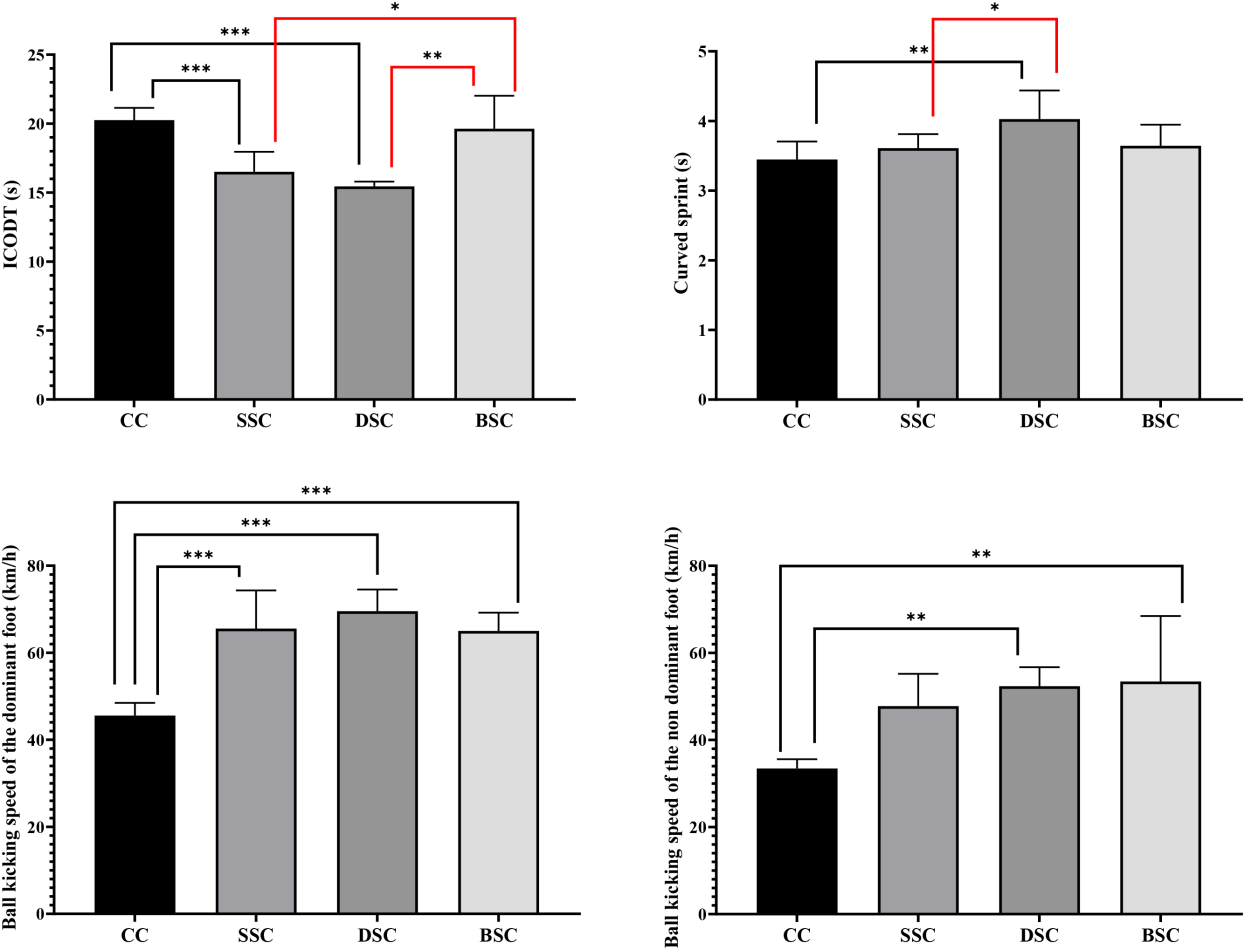
Agility, curved sprint speed, and ball kicking speed depending on warm-up conditions. ICODT, Illinois Change of Direction Test; CC, control condition; SSC, static stretching condition; DSC, dynamic stretching condition; BSC, ballistic stretching condition. *: p < 0.05. **: p < 0.01. ***: p < 0.001.

However, in ICODT with recorded in the DSC (15.4 ± 0.34 s) and SSC (16.5 ± 1.45 s) compared to the CC (20.2 ± 0.89 s), with large effect sizes (ES = 7.12 and ES = 3.07, respectively). The BSC (19.6 ± 2.39 s) showed values closer to the CC. In turn, significant differences were detected in the curve sprint speed (F_(3, 32)_ = 5.88; p = 0.003), with the DSC being the best time observed (4.02 ± 0.41 s). Significant differences were found in ball kicking speed with the dominant foot (F_(3, 32)_ = 32.6; p < 0.001). The highest values corresponded to DSC (69.5 ± 4.97 km/h) and SSC (65.5 ± 8.76 km/h), significantly exceeding CC (45.5 ± 2.92 km/h), with high effect sizes (ES = 1.70-5.38). Both DSC (52.3 ± 4.38 km/h) and BSC (53.4 ± 12.0 km/h) were significantly greater than CC (33.8 ± 2.40 km/h) with the non-dominant foot, with large effect sizes (ES = 2.26-5.23) yielding significant differences (F_(3, 32)_ = 6.17; p = 0.002). These results are presented graphically in **Figure 4**.

## 4 Discussion

This study aimed to assess and compare the effects of a conventional warm-up and warm-ups including stretching exercises (SSC, DSC, and BSC) on jump performance, curve sprint speed, ICODT, and ball kicking speed in male children soccer players. The main findings revealed: (i) no significant differences in jump performance (CMJ, SJ, DJ) across any of the warm-up conditions; (ii) significant differences in curve sprint speed between SSC and DSC, as well as between DSC and CC; (iii) significant differences in the ICODT across conditions, particularly between SSC and BSC, DSC and BSC, and both SSC and DSC compared with CC; and (iv) significant improvements in ball kicking speed with the dominant foot for SSC, DSC, and BSC compared with CC, and with the non-dominant foot for DSC and BSC compared with CC.

### 4.1 Jump performance

No significant differences were observed in jump performance across the CMJ, SJ, and DJ tests, indicating that none of the warm-up conditions elicited a statistically superior acute effect on vertical jump performance in children’s soccer players. These findings are similar to those of Hernandez-Martinez et al. (2023), who also reported no significant differences in CMJ performance when comparing warm-up conditions including SSC, DSC, BSC, and a conventional warm-up in Chilean youth amateur soccer players. In contrast, López Mariscal et al. (2021) compared the effects of warm-ups with SSC and DSC at different volumes (<10 s and >10 s), reporting significant improvements (p < 0.05) in both CMJ and SJ in U16 and professional adult soccer players. Conversely, Oliveira et al. (2018) observed small decreases in CMJ performance following SSC, DSC, and BSC in youth soccer players compared to a conventional warm-up, reinforcing the inconsistency of acute stretching effects on jumping tasks in younger populations.

These discrepancies may be partly explained by age-related neuromuscular characteristics. Children exhibit a lower capacity for voluntary motor unit recruitment and reduced musculotendinous stiffness compared with adolescents and adults (Haugen et al., 2014; Pietraszewski et al., 2021), as well as greater muscle–tendon compliance and less mature tendon mechanical properties, which may limit their ability to acutely enhance force production and jump performance following warm-up interventions. Although children present greater connective tissue compliance and neural plasticity (Sanati et al., 2022), the lower passive stiffness and reduced capacity to increase musculotendinous stiffness acutely may attenuate the potentiation effects typically associated with stretching-based warm-ups. Consequently, these characteristics do not necessarily translate into immediate improvements in maximal vertical jump tasks, particularly when performance is primarily constrained by neuromuscular activation capacity rather than elastic energy reutilization (Wang et al., 2024). Moreover, the immature neuromuscular system in pre-pubertal children may further limit the transfer of acute stretching-induced changes to explosive tasks, reinforcing the notion that stretching responses at this stage of development differ qualitatively from those observed in adults.

In the present study, although effect size analysis revealed moderate to large effects for some pairwise comparisons (i.e., CMJ: CC vs. DSC), these magnitudes should be interpreted with caution, as they were not accompanied by statistically significant differences. Therefore, these effects cannot be interpreted as evidence of a clear performance advantage of DSC or other stretching modalities in jump performance. Instead, these effects are more likely to reflect substantial interindividual variability in acute warm-up responsiveness, a characteristic that is especially evident during childhood due to ongoing neuromuscular development (Donti et al., 2022; Santuz and Akay, 2023). Taken together, our findings indicate that, contrary to our initial hypothesis, DSC did not confer a superior advantage in jump performance compared with SSC, BSC, or a CC. While DSC may enhance neuromuscular readiness in tasks such as agility or ball kicking speed, its effects do not appear to translate consistently to vertical jump performance in children. Consequently, no specific stretching modality can be recommended over others for the purpose of acutely enhancing jump performance in this population, and warm-up selection should be guided by the primary performance demands of the subsequent activity (Lloyd and Oliver, 2012).

### 4.2 Curve sprint speed

Significant differences were identified in curve sprint speed, showing positive effects in CC and SSC compared to DSC, contrary to our initial hypothesis. These findings are in line with those of López Mariscal et al. (2021), who compared warm-ups including SSC and DSC at different volumes (<10 s and >10 s) and reported significant improvements (p < 0.05) in linear sprint speed in U16 and professional soccer players. Similarly, Vasileiou et al. (2013) observed that DSC significantly improved 20 m sprint speed (p < 0.05), with decreases of 0.02–0.06% compared to SSC in amateur soccer players. In contrast, Hernandez-Martinez et al. (2023) found no significant differences in 10, 20, and 30 m linear sprint speed when comparing SSC, DSC, BSC, and a conventional warm-up in Chilean children soccer players. This discrepancy may be explained by the nature of the test, while Hernandez-Martinez et al. (2023) assessed straight-line sprinting, our study examined the curve sprint speed, a task that imposes greater demands on intermuscular coordination and the utilization of the stretch–shortening cycle in multidirectional trajectories (Haugen et al., 2014; Pietraszewski et al., 2021).

The discrepancy between these findings and those of previous studies may be explained by the specific biomechanical and coordinative demands of curvilinear sprinting, which differ substantially from straight-line sprinting. Curve sprint speed requires the simultaneous production of propulsive and centripetal forces, greater intermuscular coordination, and asymmetric force application between limbs (Lees et al., 2010; Rađa et al., 2019; Zago et al., 2014). In this context, the neuromuscular demands of curve sprint speed may not benefit from the acute effects of DSC to the same extent as other tasks, particularly in children, whose neuromuscular system is still developing. Contrary to our initial hypothesis, DSC did not improve curve sprint speed performance and was associated with slower times compared to CC and SSC. This finding suggests that the transient increases in neuromuscular activation and muscle-tendon compliance induced by DSC may not be advantageous for tasks requiring high levels of directional control, limb stiffness, and force redirection, such as curvilinear sprinting. In contrast, the CC and SSC conditions may better preserve musculotendinous stiffness and motor control, which are critical for maintaining sprint efficiency along curved trajectories (Zago et al., 2014).

From a neuromechanical perspective, while dynamic stretching has been shown to increase intramuscular temperature, calcium kinetics, and muscle spindle sensitivity (Daneshjoo et al., 2024; Masugi et al., 2017), these acute effects do not necessarily translate into improved performance in all sprint modalities, particularly those involving complex force-vector orientation. Therefore, the inferior performance observed following DSC in the present study highlights the task-specific nature of warm-up responses in children, rather than a consistent benefit of dynamic stretching across different performance tasks (Masugi et al., 2017). Taken together, these results indicate that DSC should not be prioritized when the primary objective is to optimize curve sprint speed performance in children’s soccer players, whereas CC and SSC appear to be more suitable warm-up strategies for this specific task. SSC, when applied in moderate volumes, may contribute to joint mobility without compromising the mechanical requirements of curve sprint speed, supporting its strategic inclusion depending on the performance context.

### 4.3 Agility

Significant differences were found in the ICODT for both DSC and SSC compared to the CC, with DSC showing a slight superiority over SSC (+6.66%). Our findings are consistent with previous reports in both young and adult soccer players, where DSC has been associated with improved performance in agility and change-of-direction tasks (Amiri-Khorasani et al., 2010; Gelen, 2010). From a physiological perspective, DSC may enhance performance in such tasks by increasing neuromuscular excitability and intermuscular coordination, thereby enabling more efficient transitions between acceleration, deceleration, and re-acceleration during directional changes. In turn, the application of SSC in moderate volumes (≤30 s) may have contributed to improved postural control and range of motion at extreme joint positions, which is beneficial for executing short displacements and pivoting maneuvers. The improvement in ICODT after dynamic stretching might also be linked to enhanced neuromuscular readiness and proprioceptive activation (Sanati et al., 2022; Wang et al., 2024). Dynamic movements stimulate the γ-motor neuron system, increasing muscle spindle sensitivity and joint position awareness (Santuz and Akay, 2023) key determinants for efficient braking and re-acceleration phases during change-of-direction tasks. These mechanisms could be particularly relevant in children, whose motor control and sensory integration systems are still developing, allowing dynamic warm-ups to act as neuromotor primers before complex agility tasks.

Moreover, it should be considered that children soccer players (hereafter referred to as children) exhibit greater musculotendinous plasticity and a critical window for flexibility development (Donti et al., 2022), which could potentiate the effects of DSC by facilitating elastic energy reutilization and improving the efficiency of the stretch–shortening cycle during change-of-direction actions. Furthermore, it has been reported that dynamic warm-ups at these ages also provide a pedagogical opportunity to consolidate fundamental movement patterns (running, braking, turning, accelerating), which not only enhance immediate performance but also contribute to long-term athletic development (Lloyd and Oliver, 2012). Overall, the present results indicate that DSC could represent a suitable approach for enhancing agility performance in children, whereas SSC, when applied in moderate volumes, could be integrated into warm-up design for complementary purposes, particularly in technical preparation and the development of functional mobility.

### 4.4 Ball kicking speed

Significant differences were found in the ball kicking speed test with the dominant foot across all stretching conditions compared to the CC. In contrast, for the non-dominant foot, significant improvements were observed only after DSC and BSC compared to the CC. Similarly, Amiri-Khorasani et al. (2010) reported increases in ball kicking speed following DSC (5.1%) and SSC (6.3%) compared to the CC in young soccer players. In addition, Gelen (2010) observed significant improvements (p < 0.05) in ball kicking speed (+3.3%) after BSC in professional soccer players. These findings differ from those of Hernandez-Martinez et al. (2023), who reported no significant differences in ball kicking speed with either the dominant or non-dominant foots when comparing SSC, DSC, BSC, and a conventional warm-up in Chilean children soccer players. A possible explanation for the larger effect magnitudes observed in the present study compared with our previous work may relate to contextual factors such as competitive season timing, technical automatization, and readiness to express performance. However, this interpretation should be considered speculative, as these factors were not directly measured and cannot be established as causal based on the present data. In the study by Hernandez-Martinez et al. (2023), assessments were conducted during the preseason or early training phase, a period typically characterized by higher training loads, residual fatigue, and less stabilized technical execution. In contrast, the present study was conducted during the competitive season, when players exhibit greater technical automatization, neuromuscular efficiency, and readiness to express performance acutely.

In this context, an increase in ball kicking speed may be considered a key performance variable, as faster shots increase the likelihood of scoring by reducing the goalkeeper’s or opponent’s reaction time (Rađa et al., 2019). Although the timing of impact and the quality of kicking technique are crucial, our findings suggest that DSC and BSC should be prioritized to optimize ball kicking speed in both foots, while SSC may serve a complementary role depending on the player’s technical development. However, it is important to consider that differences between the dominant and non-dominant foots may be more closely related to technical factors such as the quality and automatization of the kicking gesture than to the type of warm-up itself (Lees et al., 2010). Moreover, in the case of children, limited sporting experience, motor control asymmetry, and the ongoing process of technical learning may amplify these differences, making the response to stretching modalities dependent not only on the type of stretching applied but also on the level of technical mastery of each foot (Lees et al., 2010; Zago et al., 2014). From a neuromuscular perspective, DSC and BSC may transiently potentiate agonist muscle activation and intermuscular coordination, enhancing the timing and velocity of limb acceleration during the kicking motion (Daneshjoo et al., 2024). The improvement observed in non-dominant foot performance following DSC and BSC could be attributed to increased neural excitability and reduced reciprocal inhibition (Masugi et al., 2017), facilitating more synchronized movement of hip flexors, knee extensors, and plantar flexors during ball impact. This aligns with the concept of post-activation potentiation, where prior dynamic contractions enhance subsequent explosive motor output.

Finally, the large improvements observed in the present study should be interpreted within the context of the players’ competitive phase, highlighting that the acute effects of stretching-based warm-ups on ball kicking speed performance are highly sensitive to training status and seasonal timing. This interpretation aligns with the concept that warm-up responses are not only task-specific but also context-dependent, particularly in developing athletes.

### 4.5 Limitations and strengths of the study

Our study presents the following limitations: (i) no neurophysiological measurements (e.g., electromyography or neural activity) were included, which would have allowed for a more precise explanation of the mechanisms underlying muscle activation in response to different stretching modalities; (ii) the sample size was relatively small and consisted exclusively of male participants, which limits the generalizability of the results to female soccer players and other age groups, and therefore caution should be exercised when applying these findings; (iii) biological maturation status was not directly assessed. Given the age range of the participants (9–13 years), it is likely that the sample included children at different stages of pubertal development, which may have contributed to inter-individual variability in neuromuscular responses to the warm-up conditions. In addition, although the selected outcome measures are commonly used in youth soccer research, several have been primarily validated in adolescent or adult populations, which may limit their sensitivity in pre-pubertal children. Furthermore, the stretching durations applied in the present study (30 s per exercise) were derived from protocols commonly used in adult and youth literature; however, their physiological appropriateness and attentional suitability for children have not been conclusively established. Finally, the large effect sizes observed for some outcomes should be interpreted with caution, as crossover designs with relatively homogeneous samples may inflate effect magnitudes due to low within-subject variability. Collectively, these limitations restrict the generalizability of the findings but do not compromise the internal validity of the randomized crossover design. Future studies should incorporate objective indicators of biological maturation, child-specific validation of outcome measures, and age-appropriate stretching prescriptions to better contextualize acute performance responses in children’s soccer players.

On the other hand, the study also presents important strengths: (i) the randomized crossover design of the warm-up conditions, which helped minimize interindividual bias and increased the internal validity of the findings; (ii) the choice of stretching protocols (SSC, DSC, and BSC) representative of those most commonly used in soccer practice, enhancing the applied relevance of the results; and (iii) the inclusion of a diverse battery of tests (CMJ, SJ, DJ, ICODT, curve sprint speed, and ball kicking speed), which provided a comprehensive assessment of the transfer of stretching modalities to soccer-specific performance demands.

Future research should incorporate neurophysiological and biomechanical measurements to further elucidate the underlying mechanisms, as well as consider larger samples, include female soccer players, and explore different competitive levels and age groups. Additionally, examining the optimal combination of stretching modalities and the appropriate duration of warm-up protocols would be valuable to maximize their impact on performance in both developmental and high-performance contexts.

### 4.6 Practical applications

With respect to jump performance, no statistically significant differences were observed between warm-up conditions in CMJ, SJ, or DJ. Therefore, no specific stretching modality can be recommended for the purpose of acutely enhancing vertical jump performance in children’s soccer players. Although some stretching modalities showed small to moderate effect sizes in isolated comparisons, these findings should be interpreted with caution and do not support a clear performance advantage. From a practical perspective, DSC, SSC, and BSC may be considered neutral strategies for jump performance when applied in short durations, as none of the warm-up conditions induced statistically significant changes in jump performance. Regarding change-of-direction performance, both DSC and SSC significantly improved ICODT compared to CC, with DSC showing slightly greater effects. Accordingly, coaches may consider prioritizing DSC when the primary objective of the warm-up is to enhance agility and change-of-direction ability in children’s soccer players, while SSC may represent a valid alternative when applied in moderate volumes. In contrast, for curve sprint speed, superior performance was observed following the CC and SSC conditions compared to DSC. This finding indicates that DSC should not be prioritized when the main goal is to optimize curve sprint speed performance in children, and that CC or SSC may be more appropriate for this specific task. For ball kicking speed, stretching-based warm-ups demonstrated clear task-specific benefits. Improvements were observed for the dominant foot across all stretching conditions, whereas for the non-dominant foot, significant benefits were evident only after DSC and BSC. Accordingly, DSC may be considered when the aim is to optimize ball kicking speed in both foots, while SSC may be sufficient for the dominant foot and BSC may be particularly useful for supporting coordination and stiffness regulation in the non-dominant foot.

Overall, the present findings highlight that the effectiveness of warm-up strategies in children’s soccer players is highly task-specific. Rather than prioritizing a single stretching modality across all performance outcomes, coaches should select warm-up components based on the primary physical demands of the subsequent activity, the competitive context, and the developmental characteristics of the players.

## 5 Conclusion

Warm-ups involving stretching led to better performance in ball kicking speed dominant foot, compared to a conventional warm-up. However, only in DSC and BSC was there better performance compared to CC in the non-dominant foot. In curve sprint speed, CC and SSC led to better performance than DSC, while in ICODT, SSC and DSC led to better performance in children’s soccer players. Jump performance showed no differences between warm-up conditions. These findings are important for coaches’ decision-making in sports training for children’s soccer players. Future studies should directly quantify contextual variables such as seasonal timing, training load, and biological maturation to better explain variability in the magnitude of acute warm-up effects in children.

## Data Availability

The raw data supporting the conclusions of this article will be made available by the authors, without undue reservation.
